# Supplementary figures and images for: Unraveling the functional role of the orphan solute carrier, SLC22A24 in the transport of steroid conjugates through metabolomic and genome-wide association studies
Source: PLoS Genet. 2019 Sep 25;15(9):e1008208. doi: 10.1371/journal.pgen.1008208 (PMC6760779; doi:10.1371/journal.pgen.1008208)

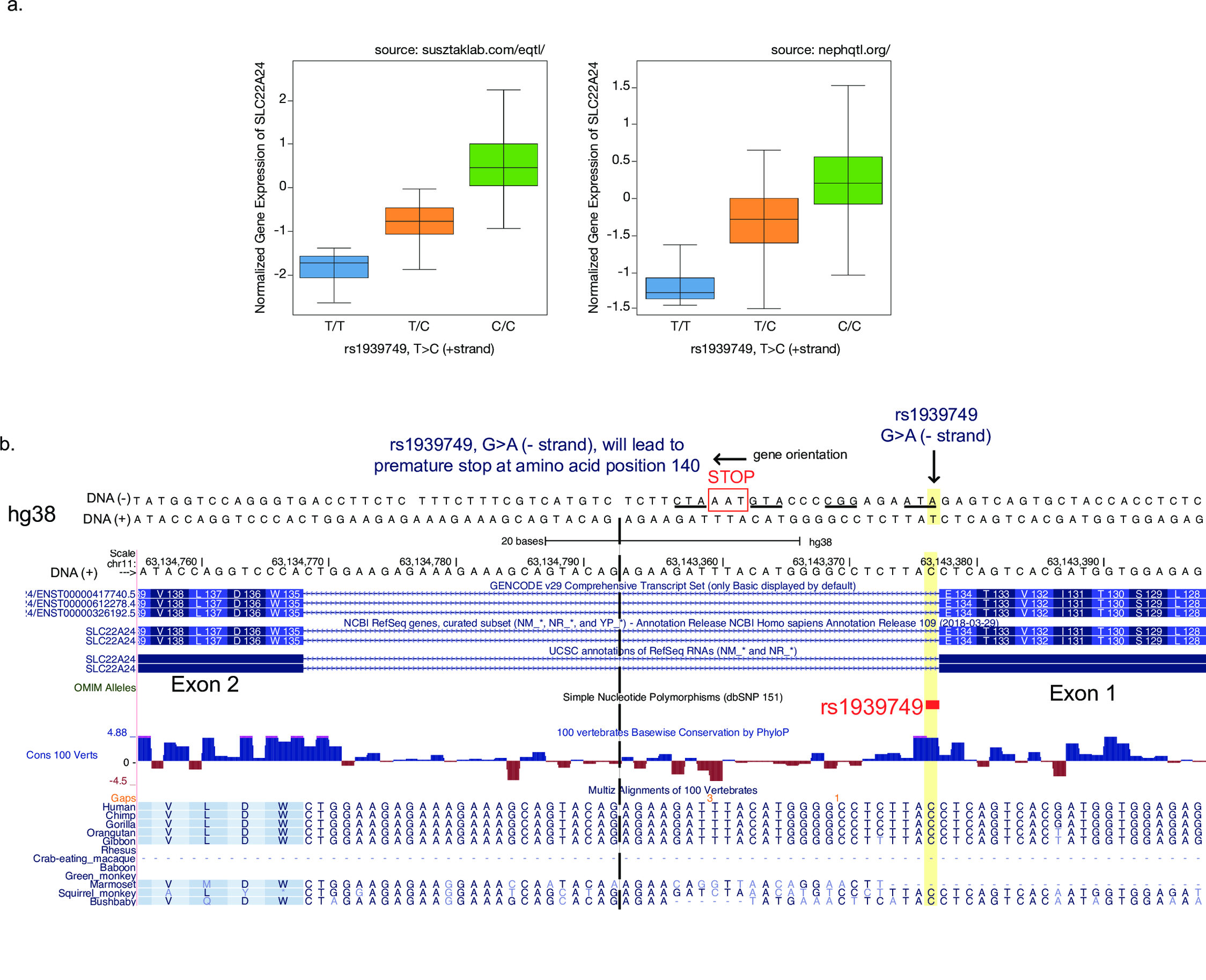

Supplement: S1 Fig — (A) A splice donor, rs1939749, C>T (+ DNA strand), is strongly associated with lower expression levels of SLC22A24 in kidney tubular cells observed in two independent kidney eQTL databases, http://susztaklab.com/eqtl and http://nephqtl.org/, p-values 3.7x10^-23^ (beta = 1.2, N = 121) and 1.9x10^-13^ (beta = 0.79, N = 166) respectively. (B) Zoom in of the SLC22A24 gene region (hg19) flanking exon 1 and exon 2 with padding of 25 bases. This splice donor, rs1939749, C>T (+ DNA strand), creates a premature stop. (TIF) [file pgen.1008208.s001.tif]

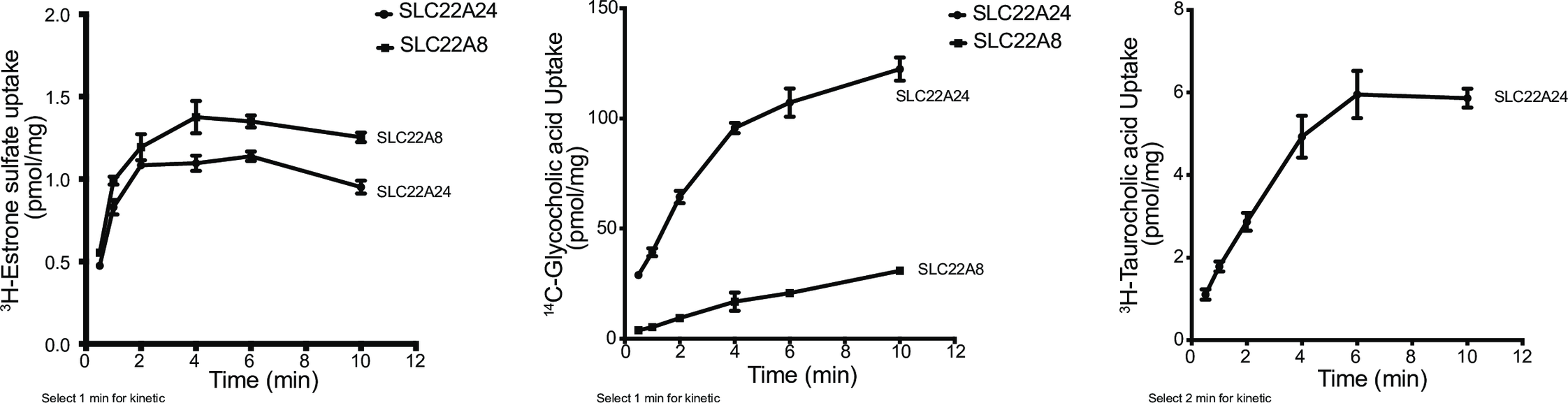

Supplement: S2 Fig — (Left) Uptake of [3H]-estrone sulfate (with 1 μM unlabeled estrone sulfate) as a function of time in cells expressing SLC22A24 and SLC22A8. The uptake rates for estrone sulfate and two other steroid glucuronides, estradiol glucuronide and androstanediol glucuronide were evaluated at 1 min. (Middle and right) Uptake of two bile acids, [14C]-glycocholic acid (with 1 μM unlabeled glycocholic acid) and [3H]-taurocholic acid (with 1 μM unlabeled taurocholic acid) as a function of time in cells expressing SLC22A24 and SLC22A8 (only glycocholic acid). The uptake rate for glycocholic acid and taurocholic acid were evaluated at 1 min and 2 min respectively. Each point represents the uptake in SLC22A24- or SLC22A8-expressing cells minus that in empty vector cells. Underlying data are provided in S1 Data. (TIF) [file pgen.1008208.s002.tif]

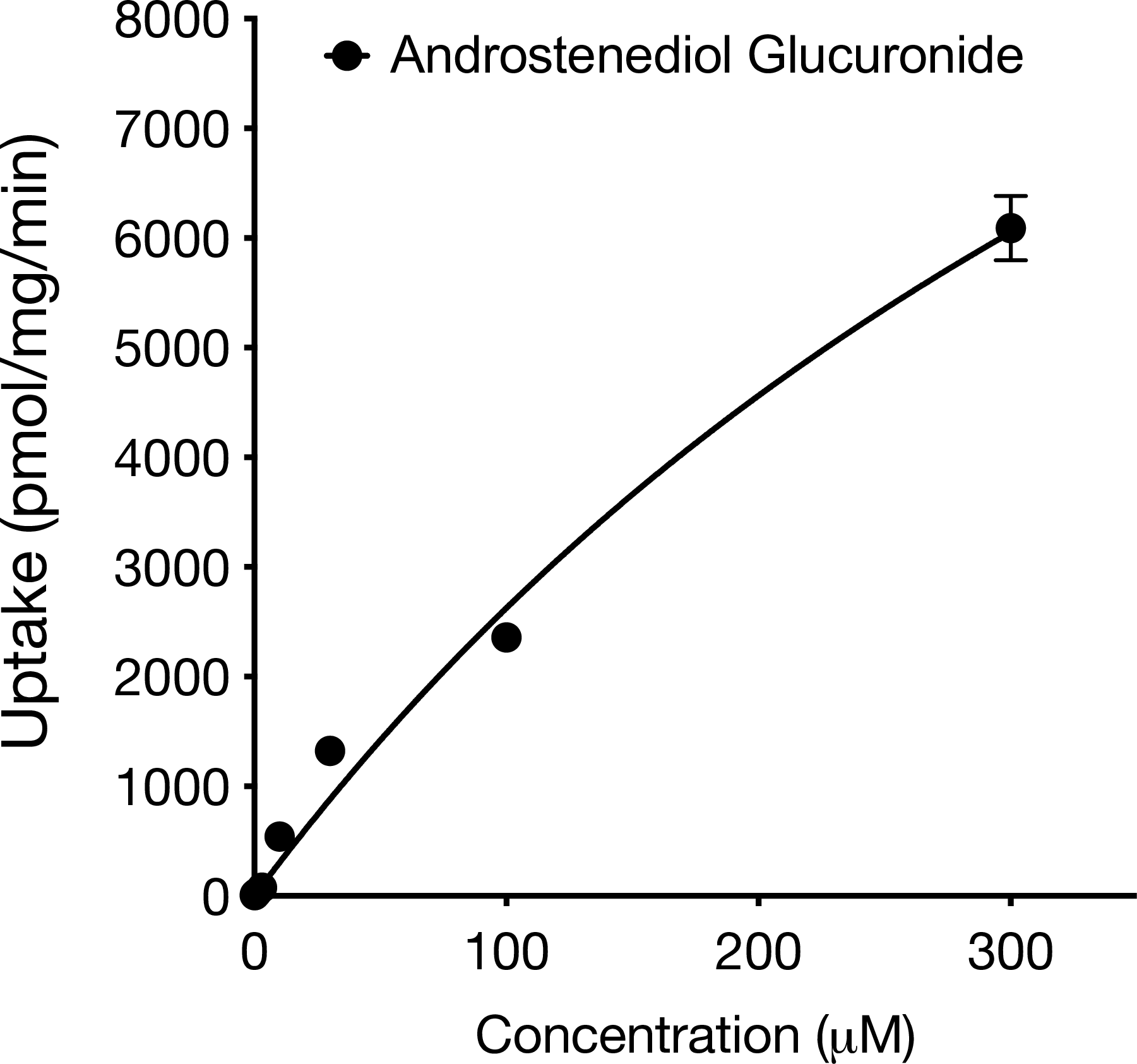

Supplement: S3 Fig — The uptake rate was evaluated at 2 minutes. Each point represents the mean ± S.D. uptake in the SLC22A24-stably transfected cells minus that in empty vector cells. Figure shows a representative plot from one experiment. The kinetic parameters of androstanediol-3-glucuronide could not be determined accurately due to a solubility limit of 300 μM. Using the plateau at 300 μM as an approximation, the affinity of androstanediol glucuronide was calculated as 741 ± 253 μM. The data were fit to a Michaelis–Menten equation. Underlying data are provided in S1 Data. (TIF) [file pgen.1008208.s003.tif]

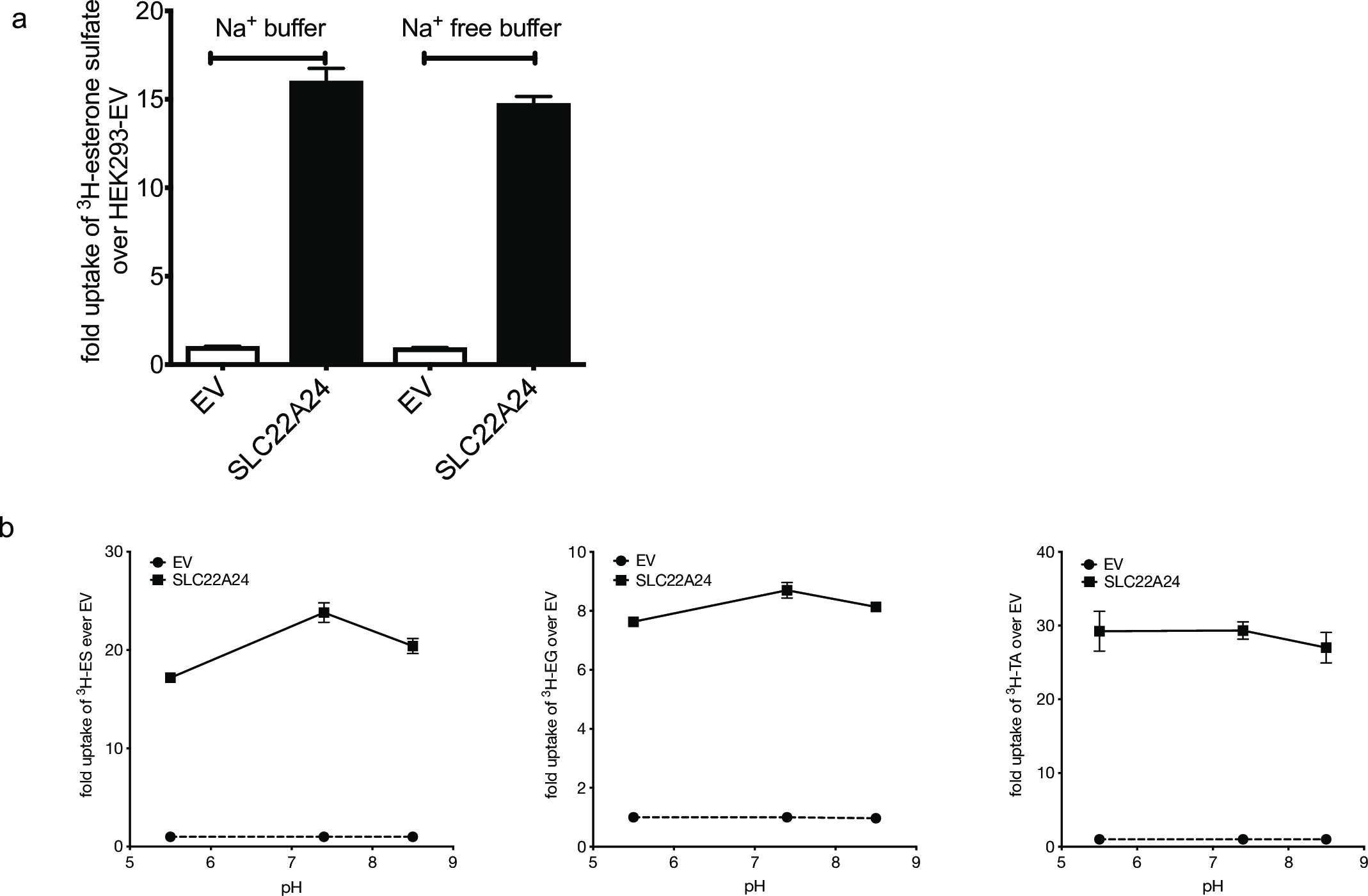

Supplement: S4 Fig — (A) [3H]-Estrone sulfate (with 1 μM unlabeled estrone sulfate) was incubated in the Na+ buffer (125 mM sodium chloride, 4.8 mM potassium chloride, 1.3 mM calcium chloride, 1.3 mM magnesium sulfate, and 5 mM HEPES, adjusted with sodium hydroxide to pH 7.4) or Na+ free buffer (125 mM choline chloride, 4.8 mM potassium chloride, 1.3 mM calcium chloride, 1.3 mM magnesium sulfate, and 5 mM HEPES, adjusted with TRIS-HCl to pH 7.4) for 10 min. Data shown are from the mean ± S.D. for representative experiments of n = 2. (B) [3H]-Estrone sulfate, [3H]-estradiol glucuronide or [3H]-taurocholic acid (with 1 μM unlabeled compound) was incubated in the HBSS buffer adjusted to pH 5.5, 7.4 and 8.5, and the cells were incubated for 10 min. Data shown are from the mean ± S.D. for representative experiments of n = 2. Underlying data are provided in S1 Data. (TIF) [file pgen.1008208.s004.tif]

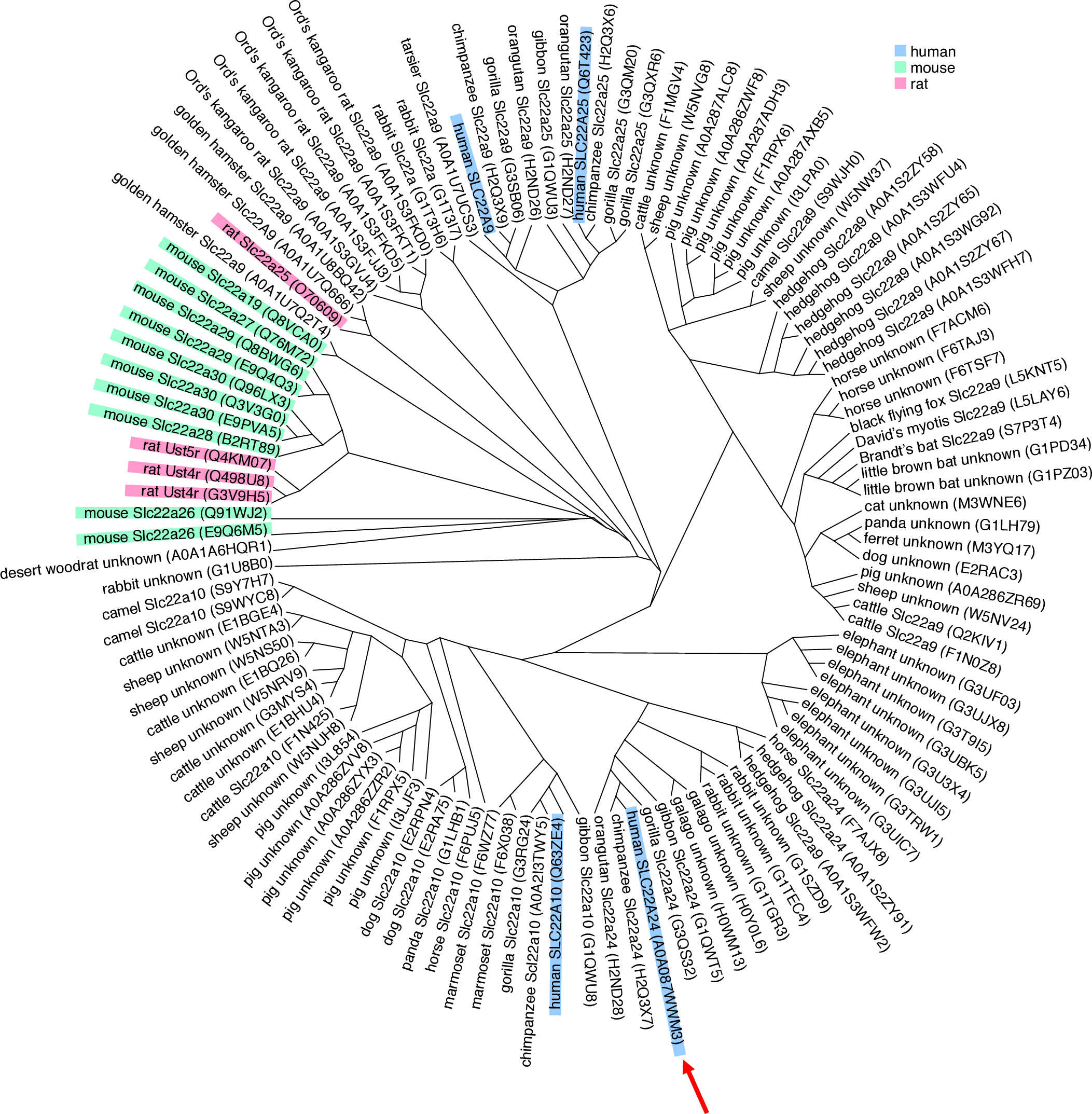

Supplement: S5 Fig — The unique and stable entry identifiers from UniProtKB (https://www.uniprot.org/) are shown in parentheses. Red arrow points to the human SLC22A24. (TIF) [file pgen.1008208.s005.tif]

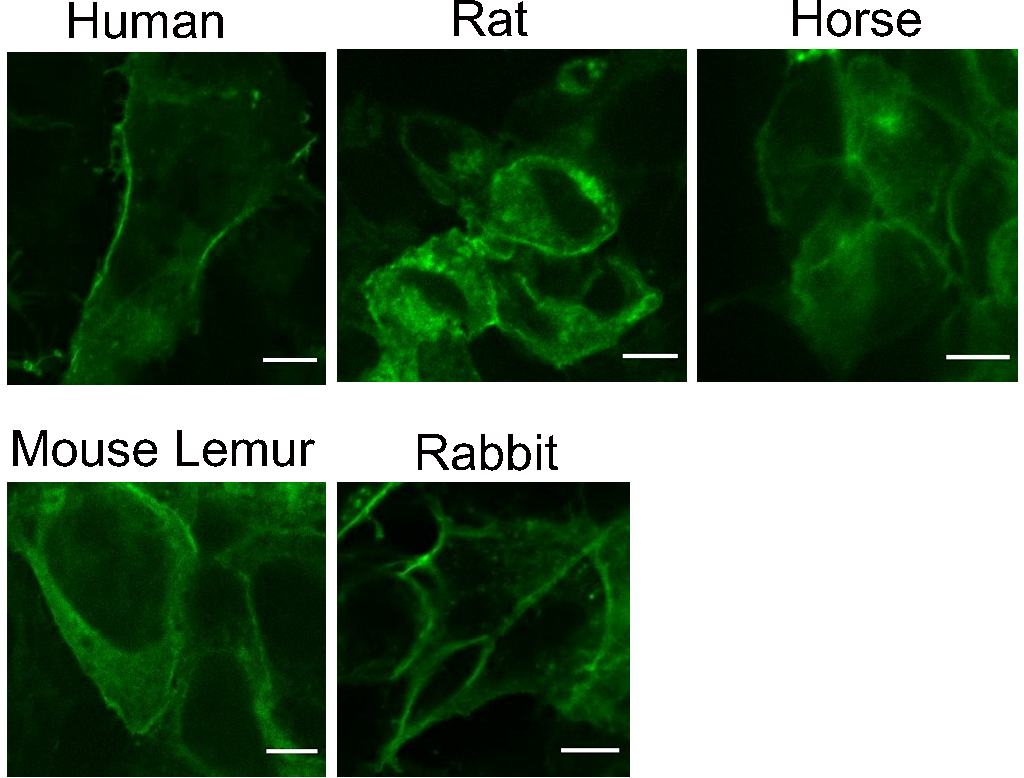

Supplement: S6 Fig — GFP fusion constructs were generated for the human, rabbit, horse, and mouse lemur SLC22A24 and the rat Slc22a9/Slc22a24. HEK293 cells were transiently transfected with the GFP fusion constructs. The cells were visualized by confocal microscopy to determine whether the GFP fused protein is expressed on plasma membrane. (TIF) [file pgen.1008208.s006.tif]

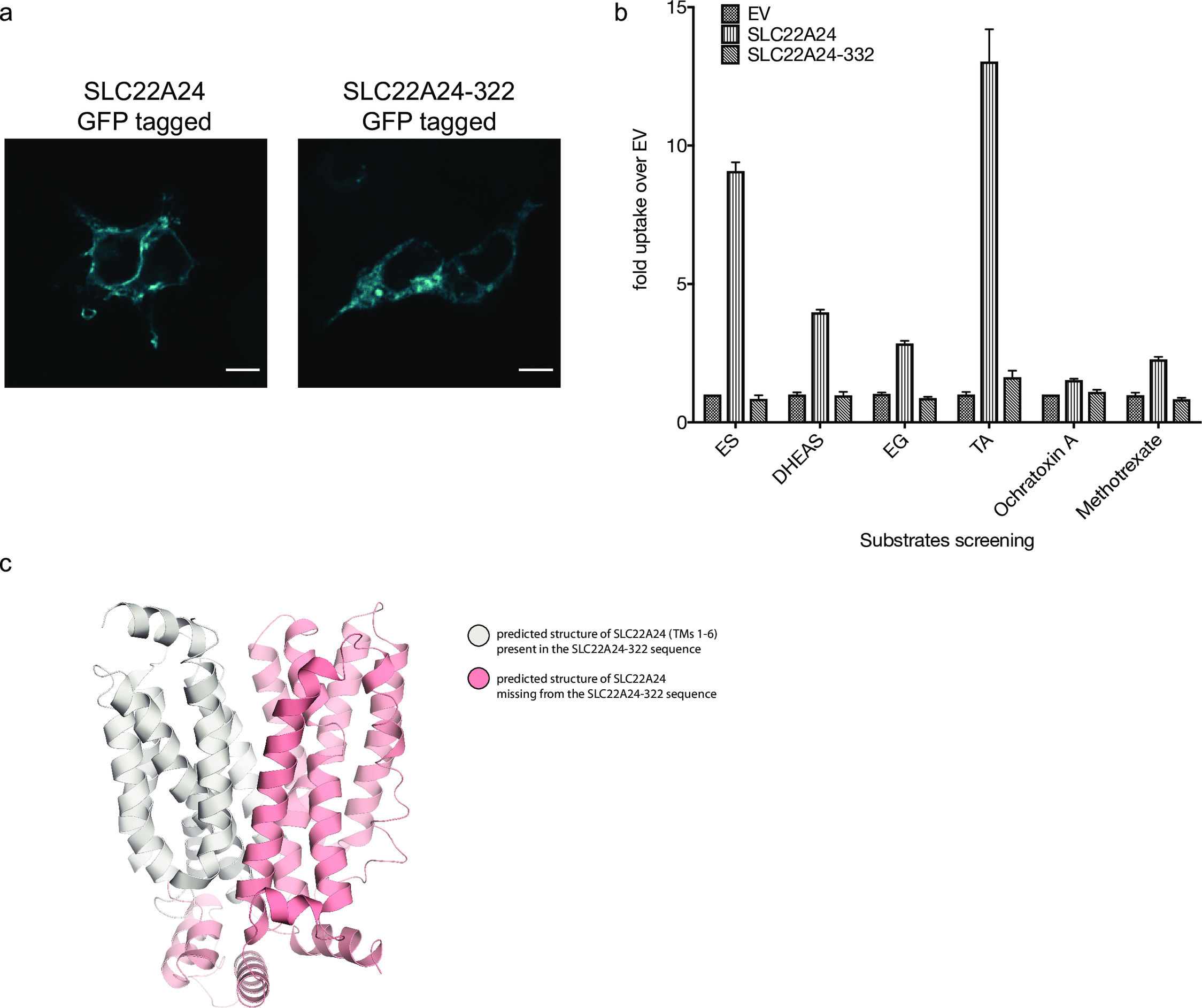

Supplement: S7 Fig — (A) GFP fusion constructs were generated for human SLC22A24 and SLC22A24-322. HEK293T cells were transient transfected with the GFP fusion constructs. The cells were visualized by confocal microscopy to determine whether the GFP fused protein is expressed on plasma membrane. (B) The uptake experiments of various anions were performed for 10 minutes in triplicate wells using trace amount of the radiolabeled compound. Experiments were repeated once. Underlying data are provided in S1 Data. (C) A comparative structure model of the human SLC22A24 with 12 transmembrane helices (white and red). SLC22A24-322 is predicted to consist of transmembrane helices 1–6 (white), but missing transmembrane helices 7–12 (red). (TIF) [file pgen.1008208.s007.tif]

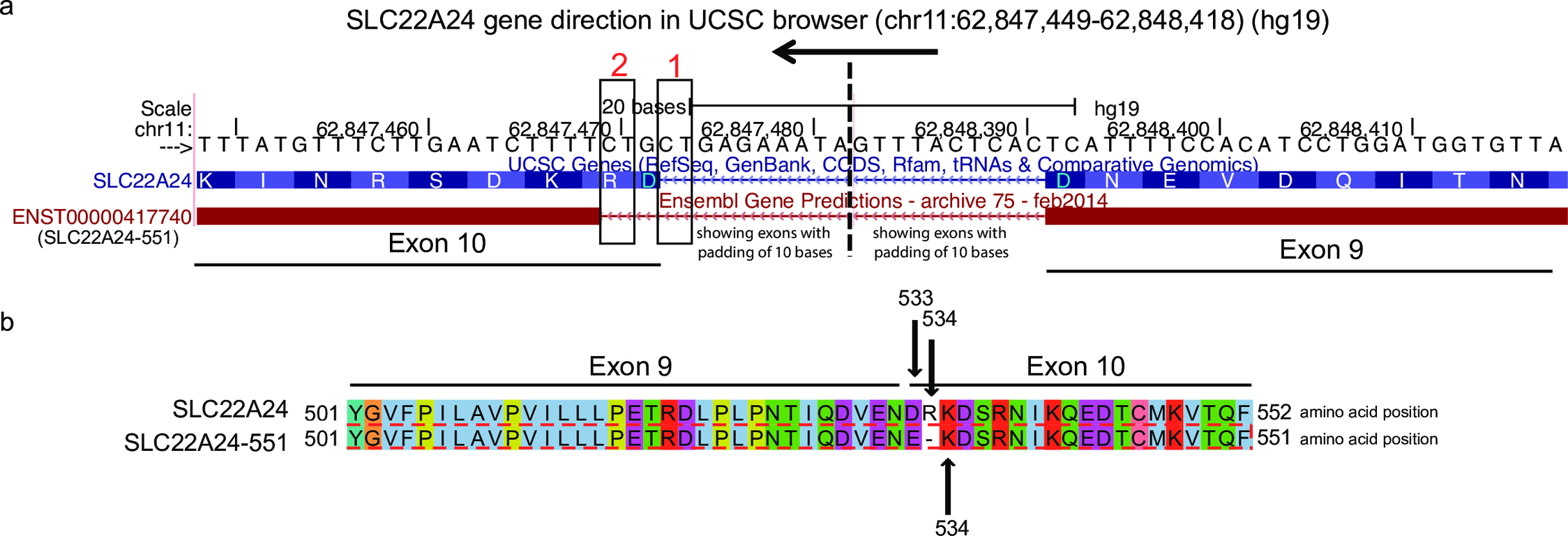

Supplement: S8 Fig — (A) Zoom in of the SLC22A24 gene region (reverse direction) between exon 9 and exon 10 along with padding of 10 bases using UCSC browser. The allele “CT” from position 1 and position 2 resulted in formation of two different isoforms (splice variants) of SLC22A24. (B) Zoom in of the SLC22A24 amino acids between exon 9 and exon 10. Arrows show the differences in amino acid positions 533 and 534, which lead to SLC22A24-551 and SLC22A24 proteins. (TIF) [file pgen.1008208.s008.tif]

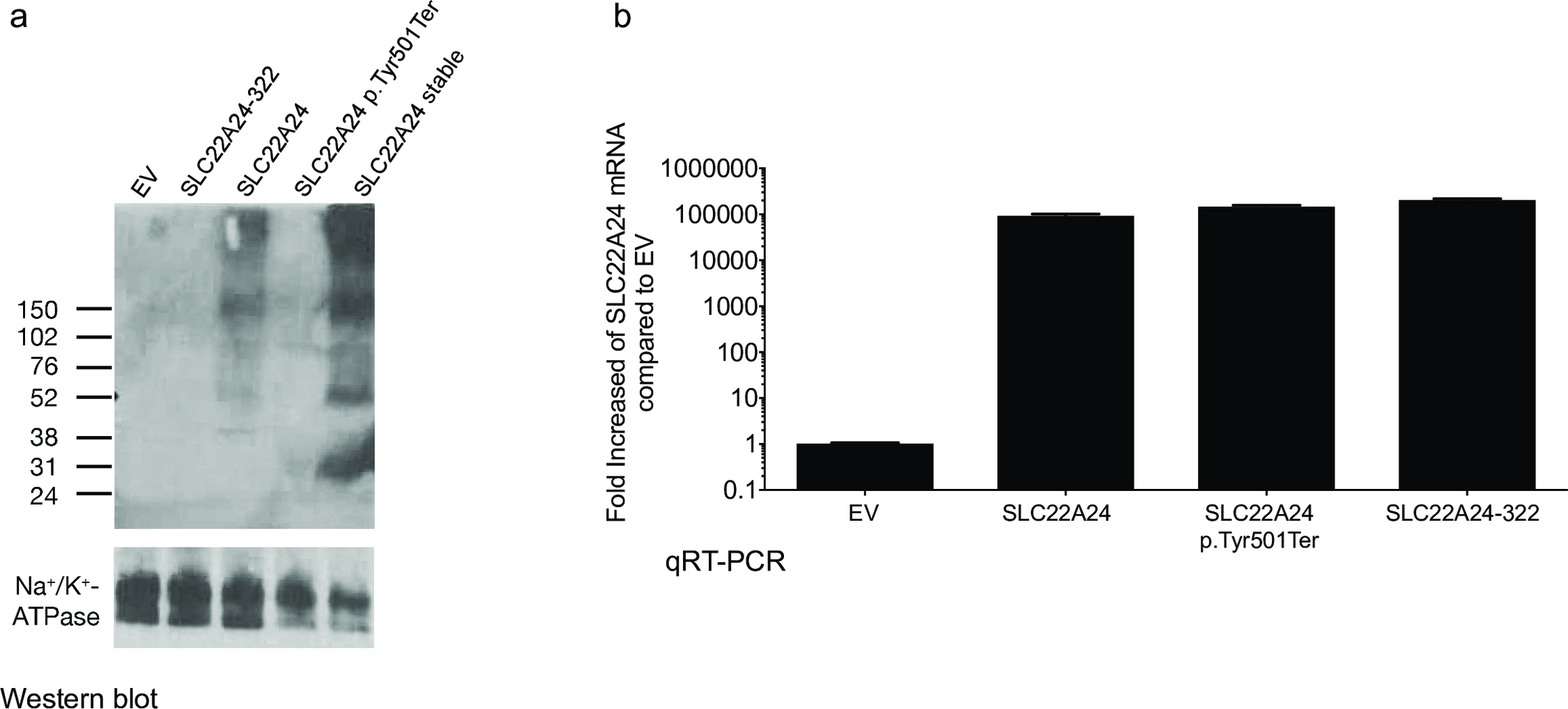

Supplement: S9 Fig — (A) Western blot of cells expressing empty vector, SLC22A24, SLC22A24 p.Tyr501Ter, and SLC22A24-322. The latter two were not expressed on the plasma membrane despite detectable mRNA levels. (B) Transcript levels of mRNA measured using Taqman assay in HEK293 Flp-In cells transiently expressing empty vector, SLC22A24, SLC22A24-322 and SLC22A24 p.Tyr501Ter. Underlying data are provided in S1 Data. (TIF) [file pgen.1008208.s009.tif]

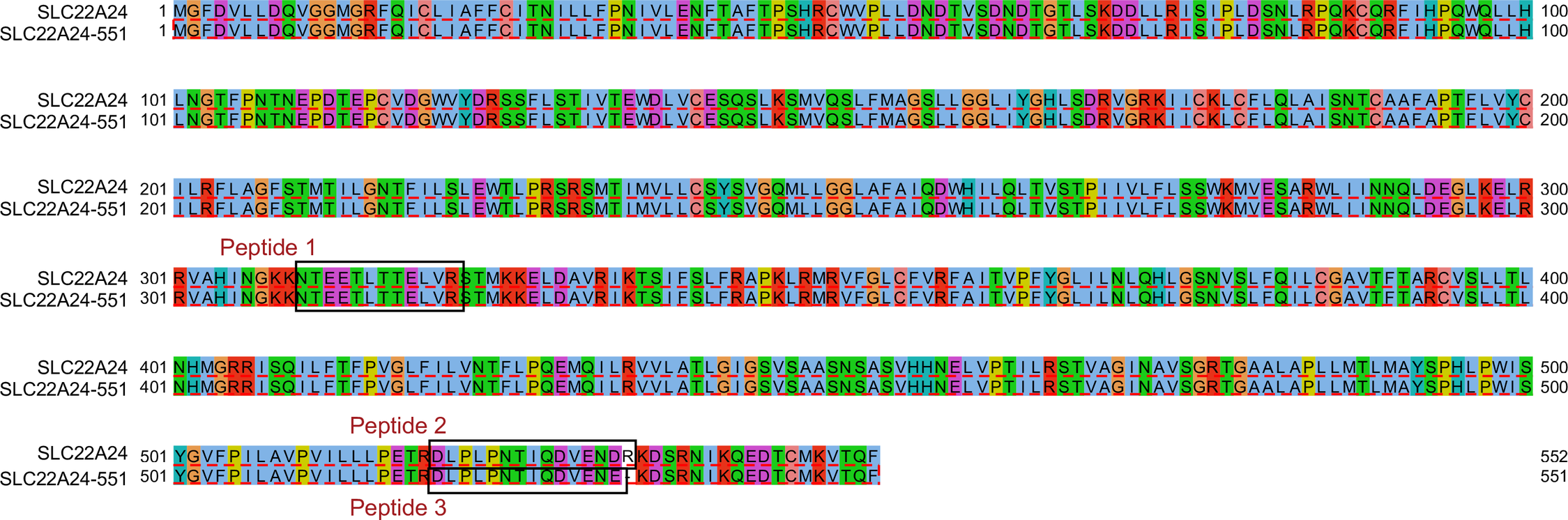

Supplement: S10 Fig — The peptides of interest and their corresponding fragments used to quantify the protein levels of SLC22A24 and SLC22A24-551 are outlined in black. Peptide 1 corresponds to the SLC22A24 and SLC22A24-551 proteins. Peptides 2 and 3 correspond to SLC22A24 and SLC22A24-551, respectively. (TIF) [file pgen.1008208.s010.tif]
