# Supplementary material for: Unraveling the functional role of the orphan solute carrier, SLC22A24 in the transport of steroid conjugates through metabolomic and genome-wide association studies
Source: PLoS Genet. 2019 Sep 25;15(9):e1008208. doi: 10.1371/journal.pgen.1008208 (PMC6760779; doi:10.1371/journal.pgen.1008208)
Supplement: S5 Table — (DOCX) [file pgen.1008208.s015.docx]

**S5 Table.** **Kinetic parameters of steroid conjugates and bile acids uptake by SLC22A24.** Kinetic parameters of the estrone sulfate, estradiol glucuronide, androstanediol glucuronide and taurocholic acid by SLC22A8 are included here as comparison to SLC22A24. The values are average ± SD from two experiments.

| Transporter: | SLC22A24 | SLC22A8 | SLC22A24 | SLC22A8 | SLC22A24 | SLC22A8 | SLC22A24 | SLC22A24 |
| --- | --- | --- | --- | --- | --- | --- | --- | --- |
| Substrate: | **Estrone sulfate** | | **Estradiol glucuronide** | | **Androstanediol glucuronide** | | **Taurocholic acid** | **Glycocholic acid** |
| Parameter |  |  |  |  |  |  |  |  |
| Km (µM) | 8.6 ± 0.3 | 7.1 ± 0.2 | 17.5 ± 0.2 | 20.0 ± 11.3 | 741.8 ± 253 | 616.0 ± 7.8 | 10.5 ± 1.1 | 33.4 ± 2.5 |
| Vmax (pmol/mg/min) | 928 ± 182 | 1241 ± 187 | 733 ± 17 | 671 ± 143 | 23204 ± 8206 | 7838 ± 52 | 751.3 ± 42 | 1722 ± 487 |
