# Supplementary material for: Unraveling the functional role of the orphan solute carrier, SLC22A24 in the transport of steroid conjugates through metabolomic and genome-wide association studies
Source: PLoS Genet. 2019 Sep 25;15(9):e1008208. doi: 10.1371/journal.pgen.1008208 (PMC6760779; doi:10.1371/journal.pgen.1008208)
Supplement: S7 Table — (DOCX) [file pgen.1008208.s017.docx]

**S7 Table**. Source of cDNA. The cDNA from two tissue panels were purchased from Takara Bio Inc. Human MTC^TM^ Panel I and II contained sixteen different tissues.

| Tissue | Pooled from how many samples | Source (Human MTC Panel I or Panel II) |
| --- | --- | --- |
| Heart | 3 male Caucasians, age 24-41 | MTC Panel I |
| Brain | 8 male Caucasians, age 43-65 | MTC Panel I |
| Placenta | 11 female Caucasians, age 19-39 | MTC Panel I |
| Lung | 1 male Caucasian, age 50 | MTC Panel I |
| Liver | 1 male Caucasian, age 35 | MTC Panel I |
| Skeletal Muscle | 4 male/female Caucasians, age 25-56 | MTC Panel I |
| Kidney | 4 male/female Caucasians, age 28-48 | MTC Panel I |
| Pancreas | 15 male/female Caucasians, age 22-69 | MTC Panel I |
| Colon with mucosal lining | 5 male/female Caucasians, age 27-61 | MTC Panel II |
| Leukocyte, peripheral blood | 550 male/female Caucasians, age 18-40 | MTC Panel II |
| Ovary | 15 Caucasians, age 20-60 | MTC Panel II |
| Prostate | 98 Caucasians, age 15-70 | MTC Panel II |
| Small intestine without mucosal lining | 32 male/female Caucasians, age 15-57 | MTC Panel II |
| Spleen | 15 male/female Caucasians, age 22-69 | MTC Panel II |
| Testis | 45 Caucasians, age 14-64 | MTC Panel II |
| Thymus | 9 male/female Caucasians, age 20-40 | MTC Panel II |
