# Supplementary material for: Unraveling the functional role of the orphan solute carrier, SLC22A24 in the transport of steroid conjugates through metabolomic and genome-wide association studies
Source: PLoS Genet. 2019 Sep 25;15(9):e1008208. doi: 10.1371/journal.pgen.1008208 (PMC6760779; doi:10.1371/journal.pgen.1008208)
Supplement: S9 Table — (DOCX) [file pgen.1008208.s019.docx]

**S9 Table**. Primers and PCR conditions used in this study. Primers used for PCR to detect SLC22A24 transcripts and to clone the SLC22A24 cDNA. PCR conditions are also provided in table below.

| **SLC22A24 transcript ID and reference sequence** | **Primer name (FW: Forward; RV: Reverse)** | **Primer sequences** | **PCR conditions (annealing temperature) and product size** |
| --- | --- | --- | --- |
| GAPDH: X01677.1 | GAPDH-FW  GAPDH-RV | TGAAGGTCGGAGTCAACGGATTTGGT  CATGTGGGCCATGAGGTCCACCAC | 57.5 °C, 30 cycles  Size: 983 bp |
| *SLC22A24 transcript variant 1: NM_001136506.2 (ENST00000417740.5 and ENST00000612278.4) | SLC22A24-551/552 FW  SLC22A24-551/552 RV | TATGGCCTGATACTCAACTTG  TTAAAACTGTGTTACTTTCATG | 57.5 °C, 40 cycles  Size: 567 bp |
| For cloning the whole gene  SLC22A24 transcript variant 1: NM_001136506.2 (ENST00000417740.5 and ENST00000612278.4) | pcDNA5-SLC22A24-BamHI_FW  pcDNA5-SLC22A24-XhoI-RV | ACCGAGCTCGGATCCATGGGCTTTGATGTGCTC  CCCTCTAGACTCGAGTTAAAACTGTGTTACTTTCATG | 57.5 °C, 35 cycles  Size: 1689 bp |
| For constructing GFP-fusion protein with the different SLC22A24 species orthologs | Horse_SLC22A24 GFP-FW  Horse_SLC22A24 GFP-RV  Mouse Lemur_SLC22A24 GFP-FW  Mouse Lemur_SLC22A24 GFP-RV  Rabbit_SLC22A24 GFP-FW  Rabbit_SLC22A24 GFP-RV  Rat_SLC22A24 GFP-FW  Rat_SLC22A24 GFP-RV  GFP-FW  GFP-RV | GAGCTGTACAAGTAACTCGAGTCTAGAGGGCCCG  GCCCTTGCTCACCATAAATTGGGTTACTTTCATGG  GAGCTGTACAAGTAACTCGAGTCTAGAGGGCCCG  GCCCTTGCTCACCATAAATTGTGTTACTTTCATGC  GAGCTGTACAAGTAACTCGAGTCTAGAGGGCCCG  GCCCTTGCTCACCATAAATTGTGTTACTTTTATGAAAG  GAGCTGTACAAGTAAACGCGTACGCGGCCGCTCGAG  GCCCTTGCTCACCATAAATGGTGTCACTTTGGCAAC  ATGGTGAGCAAGGGCGAGGAG  TTACTTGTACAGCTCGTCCATG | 57.5 °C, 35 cycles  Size: ~6.7 Kbp  57.5 °C, 35 cycles  Size: ~6.7 Kbp  57.5 °C, 35 cycles  Size: ~6.7 Kbp  57.5 °C, 35 cycles  Size: ~6.7 Kbp  57.5 °C, 35 cycles  Size: 720 bp |

Note:

*The primer set used for expression profiling of SLC22A24 transcript variant 1 is not able to distinguish 551 amino acids or 552 amino acids, however, Sanger sequencing demonstrate both forms exist at least in liver as well as kidney.
