# Supplementary material for: Unraveling the functional role of the orphan solute carrier, SLC22A24 in the transport of steroid conjugates through metabolomic and genome-wide association studies
Source: PLoS Genet. 2019 Sep 25;15(9):e1008208. doi: 10.1371/journal.pgen.1008208 (PMC6760779; doi:10.1371/journal.pgen.1008208)
Supplement: S11 Table — (DOCX) [file pgen.1008208.s021.docx]

**Supplementary Table S11**. List of renal transporters in the solute carrier (SLC) superfamily that are known to have apical membrane localization. It has been demonstrated that apical membrane localization transporters possess a PDZ-motif consensus (S/T-X-h, where X is any amino acid and h is hydrophobic amino acids (A, F, L, M, I, W, P and V)) at their C-terminal. SLC22A24 has the PDZ-domain consensus sequence and support its role on apical membrane of the kidney.

| **Seq-ID (UniprotID\|Genename_species)** | **Sequence** | **Membrane localization** |
| --- | --- | --- |
| A0A087WWM3\|SLC22A24_HUMAN | KVTQF | Has the PDZ-domain consensus similar to other known apical membrane transporter |
| O15244\|SLC22A2_HUMAN | DIPLN | basolateral |
| Q9H015\|SLC22A4_HUMAN | LITAF | apical |
| O76082\|SLC22A5_HUMAN | KSTAF | apical |
| Q4U2R8\|SLC22A6_HUMAN | EKNGL | basolateral |
| Q9Y694\|SLC22A7_HUMAN | QVQN | basolateral |
| Q8TCC7\|SLC22A8_HUMAN | GLGSS | basolateral |
| Q9NSA0\|SLC22A11_HUMAN | ESTSL | apical |
| Q96S37\|SLC22A12_HUMAN | KSTQF | apical |
| Q9Y226\|SLC22A13_HUMAN | SSTYF | apical |
| P11168\|SLC2A2_HUMAN | GATET | apical |
| Q16348\|SLC15A2_HUMAN | KKTKL | apical |
| Q14916\|SLC17A1_HUMAN | QHTRL | apical |
| O00476\|SLC17A3_HUMAN | KLTRL | apical |
| Q9Y2C5\|SLC17A4_HUMAN | TFTHL | apical |
| Q9BZV2\|SLC19A3_HUMAN | MSTKL | apical |
